# Community-Acquired Methicillin-Resistant *Staphylococcus aureus* in Hospitals: Age-Specificity and Potential Zoonotic–Zooanthroponotic Transmission Dynamics

**DOI:** 10.3390/diagnostics13122089

**Published:** 2023-06-16

**Authors:** Ahmed Alsolami, Naif Saad ALGhasab, Mohammed S. M. Alharbi, Abdelhafiz I. Bashir, Mohd Saleem, Azharuddin Sajid Syed Khaja, Dakheel F. Aldakheel, Ehab Rakha, Jabar Aziz Alshammari, Taha E. Taha, Ziyad Melibari, Yaseer H. Alharbi, Ali A. Almutlag, Kamaleldin B. Said

**Affiliations:** 1 Department of Internal Medicine, College of Medicine, University of Ha’il, Ha’il 55476, Saudi Arabia; a.alsolami@uoh.edu.sa (A.A.); ms.alharbi@uoh.edu.sa (M.S.M.A.); 2 Department of Cardiology, College of Medicine, University of Ha’il, Ha’il 55476, Saudi Arabia; n.alghasab@uoh.edu.sa; 3 Department of Physiology, College of Medicine, University of Ha’il, Ha’il 55476, Saudi Arabia; 4 Department of Pathology, College of Medicine, University of Ha’il, Ha’il 55476, Saudi Arabia; m.saleem@uoh.edu.sa (M.S.); skazharuddin@uoh.edu.sa (A.S.S.K.); j.alshammary@uoh.edu.sa (J.A.A.); s201906498@uoh.edu.sa (Z.M.); s201905835@uoh.edu.sa (Y.H.A.); s201905824@uoh.edu.sa (A.A.A.); 5 Medical Coordination Unit, Ha’il General Hospital, Ha’il 55428, Saudi Arabia; daldakheel@moh.gov.sa; 6 Departments of Microbiology, King Khalid Hospital, Ha’il 55421, Saudi Arabia; ehabrakha@yahoo.com; 7 Clinical Pathology Department, Faculty of Medicine, Mansoura University, Mansoura 35516, Egypt; 8 Department of Epidemiology, John Hopkins Bloomberg School of Public Health, Baltimore, MD 21205, USA; ttaha1@jhu.edu; 9 Genomics, Bioinformatics and Systems Biology, Carleton University, 1125 Colonel-By Drive, Ottawa, ON K1S 5B6, Canada

**Keywords:** *S. aureus* epidemiology, CA-MRSA age-specificity, geriatric-HA-MRSA, *S. aureus*-antibiogram

## Abstract

Methicillin-resistant *Staphylococcus aureus* (MRSA) lineages are a devastating clinical and public health issue. Data on local lineage profiles are limited. We report on the frequency of community-acquired and hospital-acquired cases (CA-MRSA, HA-MRSA). We studied 147 isolates from King Khalid tertiary care hospitals (KKH), each from a case in a patient and including 33 patients at the Maternity and Children’s Hospital (MCH). Of the 147 isolates, 87 males (59%) and 60 females (41%) were in KKH. The overwhelming majority (80%; *n* = 119/147) were CA-MRSA in KKH. Intriguingly, despite significant differences between males (70%) and females (53%), lineage-acquisition remained age-specific around 58–60 years in both genders. However, while CA-MRSA dominated early in life (0–20, 70% MCH), it increased with age in KKH adults; 21–50 (28%), >50 (59%) until the overall 80% (*n* = 144/180). Major specimens included skin-wounds, surgeries (70.3%), blood (13.5%), sputum (8.8%), very rarely urine (4.1%), and nasal (3.4%), albeit most patients showed severe enteritis and necrotizing pneumonia. Antibiograms showed high beta lactam resistances, including amoxicillin–clavulanate (83%), oxacillin (84%), cefoxitin FOX (100%), penicillin and ampicillin (~100%), as well as high resistance (82%) to carbapenem. Fortunately, high susceptibility was seen to non-beta lactams and, to a lesser extent, gentamicin, erythromycin, and fusidic acid; 33%, 34%, and 38%, respectively, in KKH. A similar pattern was seen in MCH except for a low resistance pattern to gentamicin CN, clindamycin CD, erythromycin E, and tobramycin TOB; 34%, 31%, 39%, and 41%, respectively, except for fusidic acid. These findings have significant clinical implications for MRSA patient management strategies. Clinical- and lineage-profiles imply host-selection and zoonotic–zooanthroponotic transmission dynamics. Future molecular typing, sequencing, and characterization of dominant clone(s) is imperative.

## 1. Introduction

The devastating emergence and re-emergence of *Staphylococcus aureus* (*S. aureus*) lineages in hospitals and communities have been significant public health concerns globally. The World Health Organization (WHO) listed MRSA lineages as a priority pathogen and labeled them as one of the indicators for antimicrobial resistance in the Sustainable Development Goals connected to the health target 3.d [1]. Since the global MRSA pandemics a decade ago, outbreaks with high morbidities and mortalities are still being reported globally not in spite of, but because of advances in medicine [2,3]. Despite enormous efforts, it is not yet clear how a species with a clonal genome continuously evolves. It has become critical to evaluate the adaptive distribution of *S. aureus* in the contexts of host factors and genome plasticity. The previously established patterns of evolutionary history in *S. aureus* across decades implies that only a handful of subtle mechanisms are responsible for the rapid emergence of host-specific lineages, despite a stable genome. We and others have previously provided solid evidence, both from core genome and highly polymorphic loci, that *S. aureus* from humans and animals has a common genomic background and gene content [4,5,6,7]. Yet, lineages continue to emerge into sub-clonal populations specifically associated with humans and animals, as we have shown earlier [8,9]. One of the major reasons why that has not been fully explored with regard to continued emergence is the human host intracellular factor, which differentiates selective expression of virulence in community-associated lineages [10]. This stems from several striking observations that expression patterns of intrinsic host factors remained the same in methicillin-sensitive and resistant *S. aureus* (MSSA and MRSA) lineages of the same genotype, supporting the idea that gene transfer and resistance alone, although they may aid in transmission, do not account for the entire virulence per se [11,12,13]. Therefore, it has become imperative to study *S. aureus* lineages in the context of both human demographics and antibiogram patterns.

*Staphylococcus aureus* is among the first described pathogens that still continues to cause serious infections and is carried in the nasal mucosa and skin surfaces. Since first isolated by Ogston A (1881) [14] from surgical wound infection, *S. aureus* continues to re-emerge with enhanced virulence. This includes skin and soft tissue infections (SSTIs) as bullous impetigo, furuncles, and cellulitis, central-line-associated bloodstream infections (CLABSI), systemic infections such as bloodstream infection (BSI) leading to sepsis, bacteremic pneumonia, and endocarditis, in addition to toxin-associated diseases (such as toxic shock syndrome and food poisoning) [15,16,17]. The source of these infections is mostly endogenous, since 20% and 30% of individuals have been well-known to carry *S. aureus* either permanently or occasionally. [18,19]. Several reasons explain why *S. aureus* is a deadly pathogen in human medicine. Lineages possess highly similar genomes that allow gene transfer and rapid host-specific adaptations by plastic accessory elements that code for a repertoire of mosaic virulence factors and antimicrobial resistance, inducing the emergence of virulent host- and organ-specific clones [4,5,6,7,8]. The evolution of virulence in these clones is initiated by genetic and cellular regulation of fitness, selection, and virulence factors in different types of host-specific microenvironments, leading to the coordinative production of several factors, including toxin production, biofilm formation, immune evasions, tissue invasion, internalization, intracellular persistence, and/or emergence of diseases [20]. This is particularly clear in the evolution of mastitis-associated *S. aureus* strains [9]. Therefore, the major reason for the dissemination of resistance has been that common ancestors differentiated into different host species, allowing the transfer of strains. In fact, only two years after the introduction of penicillin, *S. aureus* developed antimicrobial resistance [21]. Consequently, the overuse of the semisynthetic methicillin produced in late 1950s led to the emergence of methicillin-resistant *S. aureus* (MRSA) in early 1960 [22]. Then this led to waves of outbreaks by strains resistant to antimicrobials [23]. Evolutionary mechanisms continued in the same pattern throughout history, producing different types of *S. aureus* strains with unique antibiotic resistances [24,25]. For instance, the initially multidrug-resistant *S. aureus* lineage appeared to resist penicillin G, chloramphenicol, tetracycline, and erythromycin by producing penicillinase (penicillin-hydrolyzing enzyme in the mid-1940s). Since then, the widespread and heavy usage of antimicrobial agents has led to the emergence of several drug-resistant *S. aureus* strains, most notably, the MRSA lineage [26], which survives in hospitals and has adapted to so many drugs that it has been called (HA)-MRSA. It has been inferred from high-resolution evolutionary models of parsimony that MRSA has evolved a minimum of 20 times from its methicillin-sensitive *S. aureus* (MSSA) ancestor since its acquisition of the methicillin resistance determinant, which is carried on a mobile genetic element, the staphylococcal cassette chromosome *mec* (*SCCmec)* [27,28]. Most of antibiotic resistance in *S. aureus* is attributed to horizontal gene transfers of resistant cassettes as well as intrinsic chromosomal mutations [29]. In addition, a recent study revealed that the use of beta-lactams enhances MRSA virulence mechanisms controlled by the *SarA* gene family [30].

The excessive use of antimicrobials in hospitals, farms, veterinary work, and agriculture has led to the emergence of multidrug resistant strains in hospitals and communities. For example, the emergence of colistin-resistant Enterobacteriaceae occurred after the introduction of selective digestive tract decontamination in an intensive care unit [31]. Similarly, an increasing trend in the outbreaks of resistant hypervirulent Acinetobacter strains in public hospitals in southwest Iran [32], and campylobacter in broiler chickens in north Lebanon [33], are a few examples of zoonotic transmission. Perhaps one of the most devastating transmissions was that of the zoonotic and reverse-zoonotic evolutions of community-associated *S. aureus* (CA-MRSA) lineages with enhanced virulence in young and otherwise healthy individuals. As a result, waves of global pandemics with significant economic and public health losses occurred [30,34,35,36,37,38,39,40,41,42]. Many of the reasons for the emergence of virulence were due to the low-level usage of antimicrobial substances [43,44]. However, the mechanisms of CA-MRSA entailed a highly complex, orchestrated evolutionary convergence of enhanced virulence, high-resistance, and low-susceptibility to non-beta lactams [44,45,46]. In contrast, high levels of multi-drug resistance in HA-MRSA lineages have been associated with mortalities due to hospital-acquired bacteremia [47]. At present, the exchange of these lineages between communities, hospitals, and livestock animals [48] has posed challenges for planning and clinical management strategies. The most striking difference in these lineages is their clinical and antibiogram patterns in response to treatment options, which is of concern in this study. It has become so blurred that it is not clear which of the two patterns, HA-MRSA or CA-MRSA, dominates the region.

Methicillin resistance in *S. aureus* is defined as an oxacillin minimum inhibitory concentration (MIC) of greater than or equal to 4 micrograms/mL, based on antibiotic susceptibility. A standard definition of HA-MRSA refers to those isolated from in-hospital patients who tested negative to nasal screening on admission or MRSA isolated from inpatients for 48–72 h or more after their stay or after discharge [17,49,50,51]. According to epidemiology, CA-MRSA is defined as MRSA isolated from community or hospital outpatients without a recent hospitalization history and without any other known risk factors for MRSA infection. The acquisition of *mecA* or *mecC* elements classified into 13 types and carried on the staphylococcal cassette chromosome *mec* (*SCCmec*) confers resistance to most beta-lactams [52,53]. CA-MRSA strains generally harbor *SCCmec* type IV or V and are susceptible to non-beta-lactam antimicrobials. The HA-MRSA strains commonly harbor *SCCmec* types I, II, or III, which contain genes that confer broad resistance to antimicrobials [54]. These distinct differences in the antibiotic susceptibility and resistance patterns between the two lineages have become one of the most important distinguishing features. These two lineages have, as their name suggests, originated in hospitals and communities, albeit they are well-known to coexist in both settings, making it difficult to successfully diagnose and initiate an empiric therapy. This is separate from several livestock sub-lineages.

Reports on the exact frequency of HA-MRSA and CA-MRSA distributions in communities and hospitals in Saudi Arabia and Gulf countries are limited. In fact, there is a severe paucity in high quality data on rates, frequencies, and clinical pictures of infections by each lineage. A comprehensive review of articles on MRSA in the Gulf countries during 2011–2021 reported on all countries, including Kuwait (44%), Saudi Arabia (28%), and the United Arab Emirates (10%). A clear emergence in antibiotic resistance, especially against fusidic acid, ciprofloxacin, and clindamycin, was demonstrated. The regional prevalence of MRSA is reported as 25–35%, with clear dominance of community-acquired (CA)-MRSA. Panton–Valentine leucocidin (PVL)-producing strains accounted for 35–45%, with a clear association with CA-MRSA emergence, but there were some sporadic reports of the incorporation of PVL in healthcare-associated (HA)-MRSA. They reported dominant strains included EUST80, USA1100, and WA-MRSA-51. Novel strains are more likely to produce PVL and show fusidic acid resistance [55]. However, the state of these lineages in each country is not well-defined at different geographic region(s). Thus, it has become imperative to understand the patterns, distributions, demographics, clinical pictures, and potential outbreak situations in each region for local strain profiling, infection control planning, and patient management strategies. Some, although not many, widely reported cases have laid solid foundations for profiles of lineages in the region as a result of increased mortality, morbidity, and hospital stays [56]. For instance, comprehensive reports on strategic planning of the Gulf Co-operation Council Center for Infection Control (GCC-IC) have placed the emergence of antimicrobial resistance (AMR) on the top of its agenda since 2014 [57]. Consequently, a second meeting reported on the “One Health” concept from the Gulf Cooperation Council Countries (“Part Two: A Focus on Human Health”) [58]. Nevertheless, despite enormous efforts, and as is the case globally, calls for regional MRSA surveillance programs were made especially with the emergence of strains that require no underlying risk factors to cause illness, as well as the propagation of chimeric resistance elements in both HA-MRSA and CA-MRSA [55]. Since then, there have been several reports on the rise of *S. aureus*-resistant strains in the region that lacked objectivity and accuracy. However, many were useful and indicated that the rise in the isolates were predominantly from skin, soft tissue, wounds, and nasal swabs [59,60,61,62,63,64]. The antibiotics most commonly used for MRSA infections (skin and soft tissue infection) included fusidic acid, mupirocin, vancomycin, and clindamycin. Most resistances were found in the eastern region of Saudi Arabia, compared to Riyadh and other cities [59,65], while resistance has increased by three times compared to the usual rate in the UAE and Gulf countries, which have suffered higher rates of resistance [65,66,67,68,69]. For instance, MRSA genotypes with unique virulence, including a high prevalence of PVL and fusidic acid resistance in Kuwait hospitals, were reported [70,71,72].

## 2. Materials and Methods

### 2.1. Microbiology of Specimens and Antibiograms of Staphylococcus aureus Lineages from Patients

Hospital clinical and laboratory records were collected for *S. aureus* isolates, types of lineages, and antimicrobial susceptibility and patient demographics data. The major selection was based on positive specimens for *S. aureus* isolates where infections were clinically characterized in patients and specimens processed in the laboratory. All types of specimens from all patient age and gender groups were included. All records of clinical specimens from different hospital departments were processed using standard protocols from different hospital departments in Ha’il from the last quarter of 2021 to beyond the middle of 2022. Briefly, routine bacteriology and antibiograms were followed by molecular detection, characterization, and profiling of *S. aureus* lineages. Specimens were used immediately upon receipt or stored until processed. Most of the samples, such as swabs or aspirations, were subjected to processing immediately by identification on primary media and standard conditions of incubation at 37 °C for 18 to 24 h. Portions of isolate inoculums were stored immediately in liquid media at −30 °C for future downstream analysis. Automated testing and ID susceptibility on automated systems were used simultaneously. Most of this phase was performed on the BD Phoenix system (BD Biosciences, Franklin Lakes, NJ, USA) and MicroScan plus (Beckman Coulter, Brea, CA, USA). When required, sensitivities were confirmed by in vitro cultures in agar diffusions interpreted by zone interpretive standards for this region. The susceptibility testing and breakpoint interpretive standards were carried out in accordance with the recommendations of the Clinical and Laboratory Standard Institute (CLSI document M100S-26) [73].

### 2.2. Standard Definitions for Classification of Resistance in S. aureus as Multi-Drug Resistant Bacteria (MDR)

According to the recommendations of the “Center for Disease Control (CDC) and European Centre for Disease Control (ECDC)”, the following definitions are usually accepted as standard. According to Magiorakos et al. [74], the standard definition classifications considers MRSA isolates as multi drug-resistant (MDR) for their methicillin resistance and resistance to beta-lactams, except for the community-acquired lineages (CA-MRSA), since the latter is susceptible to beta-lactams. These criteria define resistances as follows: MDR = non-susceptibility to at least one agent in three or more antimicrobial categories; XDR = non-susceptibility to at least one agent in all but two or fewer antimicrobial categories (i.e., bacterial isolates remain susceptible to only one or two categories); PDR = non-susceptibility to all agents in all antimicrobial categories as reported. Known intrinsic resistances to particular drugs were not included. Thus, the MRSA criteria for defining MDR *S. aureus* classifications must include one or more of the following to apply: (1) hospital-acquired MRSA is always considered MDR by virtue of being an MRSA; (2) non- susceptibility to ≥1 agent in >3 antimicrobial categories.

### 2.3. Molecular Profiling of S. aureus Lineages by Multi-Gene Systems (GeneXpert)

The latest version of the Cepheid GeneXpert^®^ Dx system with specific all-in-one cartilages of the SA Complete and MRSA assay kits was used for the characterization and profiling of *S. aureus* isolates following the manufacturers’ recommendations and using the codes included in each kit. Depending on the kit used, assay definition files were imported into the software, such as the Blood Culture assay definition file. Then, when sample patient IDs were scanned, the kit barcodes re-populated the boxes with Assay, Reagent Lot ID, Cartridge SN, and Expiration Date. Certain types of specimens, such as sputum, require a few simple steps, such as homogenizations and mixing, before loading into cartridges. However, the general protocol for all kits is concise, automated, user-friendly, and highly robust. For instance, Xpert MRSA/SA SSTI (skin and soft-tissue infections, as well as wounds and surgical infections) swabs from deep tissue, surgical sites, and wound infections were inserted into the sample reagent vial (break-in). Then, for the vortex well, particularly for samples with mucus contents or debris, dispense the sample into Port-S, insert the cartridge, and start the test. Multi-gene primers, probes, and reagents in kits allow for robust automated direct confirmation of *S. aureus* at the species level with subsequent differentiation into *S. aureus* lineages directly from specimens. This is accomplished by the built-in primers for nuc spa, mecA, and the mec (SCCmec) gene and their direct detection from specimens utilizing automated real-time polymerase chain reaction (PCR) in a single-use, disposable, self-contained cartridge with PCR reagents inserted and inoculated directly with swabs/samples. Therefore, the influence of laboratory media that is known to trigger sensing genes on *S. aureus* is minimized, leading to adaptive genome expressions and in turn the emergence of different types. Of importance is that it reduces cross-contamination between specimens as well as cross-sequence contaminations in molecular tests. Furthermore, a sample processing control (SPC) and a probe check control (PCC) are also included. The SPC is present to control for adequate processing of the target bacteria and to monitor the presence of inhibitor(s) in the PCR reaction. The PCC verifies reagent rehydration, PCR tube filling in the cartridge, probe integrity, and dye stability. Real-time PCR assays for mecA, nuc, and lukPV were consistent as previously performed [4] using primers luk pvl-1 [gcatcaastgtattggatagcaaaagc and luk pvl-2 atcattaggtaaaatgtctggacatggtcc with TET-probe atttgtaaacagaaattacacagtta].

### 2.4. Statistical Analysis

The Statistical Package for Social Sciences software (IBM SPSS; Version 24 SPSS version 23.0 for Windows (SPSS, Inc., Chicago, IL, USA) was used. The study was descriptive and stratified analyses were conducted. In this study, we present absolute numbers, proportions, and graphical distributions. We conducted exact statistical tests for proportions and show *p*-values (based on Chi square test values) where appropriate (a *p*-value < 0.05 was considered statistically significant).

## 3. Results

**Figure 1:**
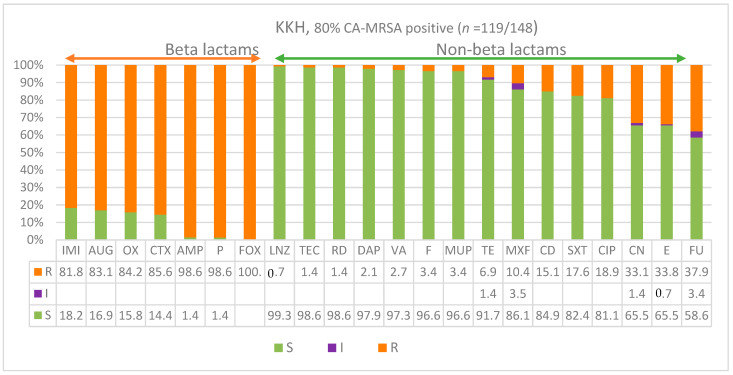
Antimicrobial susceptibility patterns of *S. aureus* isolates recovered from King Khalid Hospital across 22 antimicrobials showing (arrow heads) antibiogram patterns for beta lactam and non-beta lactam antibiotics. S = susceptible; I = intermediate resistance; R = resistance.

In the present study, we have analyzed the antibiotic-resistant pattern of MRSA obtained from different clinical specimens of 147 patients from King Khalid tertiary care hospitals (KKH) and 33 patients from the Maternity and Children’s Hospital (MCH) in the Hail region in the last quarter of 2021 and the middle of 2022. The patients’ characteristics, types of specimens, hospital wards, and antibiogram increments are all presented in Table 1. Most patients showed enteritis and necrotizing pneumonia profiles. In KKH, out of 147 patients there were 87 males (59.2%) and 60 females (41%) in our study, with 58 years being the median age of the study participants. The MCH specimens were all received and recorded under MCH samples as they all had similar descriptions that are common after birth and treated with pediatric protocol. The major types of clinical specimens targeted were those that were likely to have a skin or nasopharyngeal origin, such as skin wounds and surgeries (70.3%), blood (13.5%), sputum (8.8%), very rarely urine (4.1%), and nasal samples (3.4%). Figure 1 shows the antibiogram patterns of all non-beta lactam and beta lactam antimicrobials of isolates from King Khalid Hospital (KKH). A significantly higher number of isolates were resistant to all beta lactam antibiotics, including amoxicillin–clavulanate (83%), oxacillin (84%), cefoxitin FOX (100%), and penicillin and ampicillin (almost 100%). More important is that a higher number of isolates developed high resistance (82%) to the carbapenem antibiotic imipenem IMI. However, isolates were more susceptible to all of the non-beta lactam antibiotics with relatively lesser resistance patterns to gentamicin, erythromycin, and fusidic acid; 33%, 34%, and 38%, respectively. Furthermore, the overwhelming majority (80%; *n* = 119/148) of MRSA identified were CA-MRSA in KKH, while only 20% were HA-MRSA.

**Figure 2:**
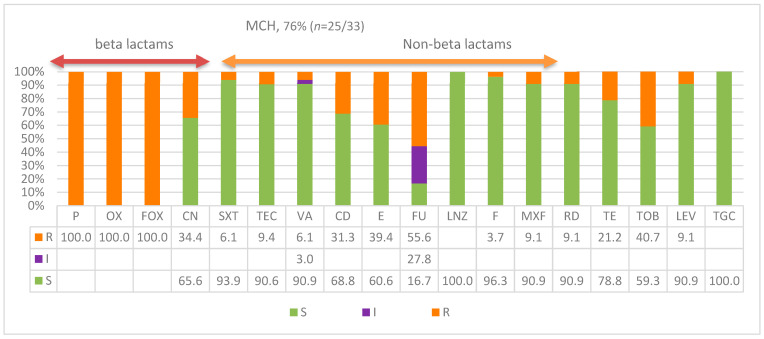
Antimicrobial susceptibility patterns of *S. aureus* isolates recovered from the Maternity and Children’s Hospital across 22 antimicrobials showing (arrow heads) antibiogram patterns for beta lactam and non-beta lactam antibiotics. S = susceptible; I = intermediate resistance; R = resistance.

Antibiogram susceptibility patterns of *S. aureus* isolates from the MCH are presented in Figure 2. Although the prescription protocol for pediatrics differs, this figure presented a much more unique pattern from that of KKH while showing similar responses of isolates to beta lactam antibiotics against which isolates were highly resistant. Isolates were fully and completely resistant to beta lactam antimicrobials used in this protocol with 100% resistance to all three: penicillin P, oxacillin OX, and cefoxitin FOX. However, similar to adult hospitals, high susceptibility of isolates was also observed for non-beta lactam antibiotics, except for low resistance patterns to gentamicin CN, clindamycin CD, erythromycin E, fusidic acid FU, and tobramycin TOB; 34%, 31%, 39%, 56%, and 41% respectively. Isolates for the rest of all non-beta lactam antimicrobials used showed a very a high pattern of susceptibility. A unique exception was the higher fusidic acid resistance compared to the MCH isolates, with higher intermediate resistance to the drug compared to all other antibiotics used in this study. In pediatrics, MCH 76% (*n* = 25/33) were CA-MRSA and 24% were HA-MRSA. Overall, 80% (*n* = 144/180) of MRSA that were CA-MRSA had the patterns.

**Figure 3:**
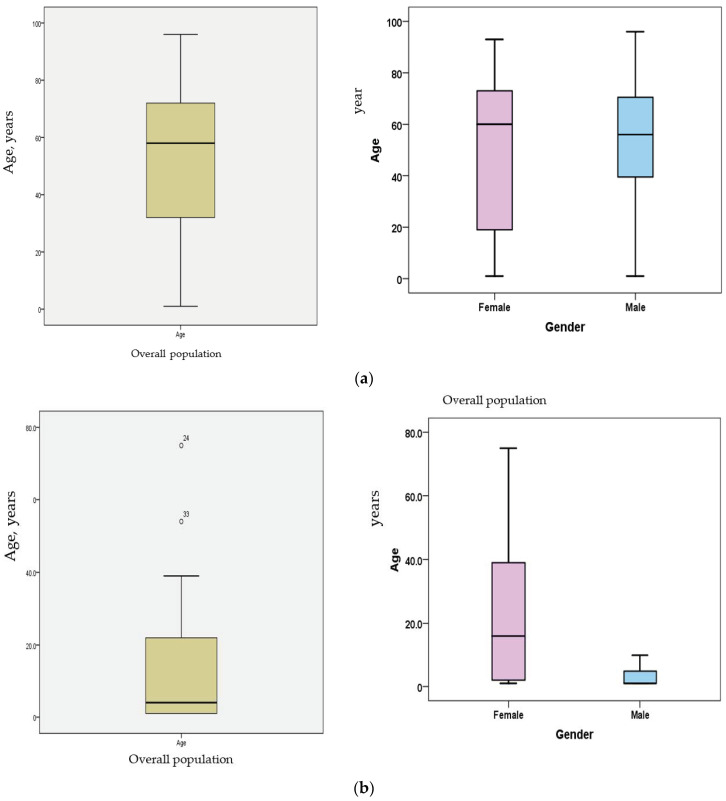
(**a**) Age- and gender-specific distributions of *Stapylococcus aureus* isolates recovered from clinical infections in King Khalid Hospital, Ha’il, Saudi Arabia. (**b**) Age- and gender-specific distributions of *Stapylococcus aureus* isolates recovered from clinical infections in Maternity and Children’s Hospital, Ha’il, Saudi Arabia.

The age- and gender-specific distributions reveal unique characteristics of potential patient groups with respect to the acquisition of specific *S. aureus* lineage infections. Males’ overall frequency (53%) was always 10% higher than that of females (43%) in contracting MRSA lineages. Most patients were in the mid age groups; the overall mean for ages was 58. The median age for male patients was 58 years while it was 60 for female patients (Figure 3a,b). The distributions of infections within these age groups in Table 1 were as follows: age groups (0 to 20) and (21 to 50) had 23% and 27% of *S. aureus* infections, respectively. While the former group included an almost similar number of isolates from MCH (*n* = 23) and KKH (*n* = 19), the latter group had the majority of isolates (*n* = 41) from KKH and only seven from MCH. The sum of isolates in these two groups constituted 50% of isolates from age 0 to 50. However, the age group of over 50 years reported 49% of isolates, which was almost equal to the sum of the isolates reported in the two younger groups. In KKH isolates, 80% (*n* = 119) that were susceptible to non-beta lactam antimicrobials confirm the CA-MRSA pattern. Of the isolates of this lineage, 49.5% (*n* = 59/119) were mostly isolated from younger age groups, from 0 to around 40 years of age, while almost all of the HA-MRSA isolates resistant to all drugs belonged to much more senior age groups of patients. Similarly, isolates of MCH were from pediatric patients and therefore, except for eight HA-MRSA, all were from children with high susceptibility to non-beta lactams. In KKH, the highest number of isolates were made on specimens received from these departments, respectively: the ICU (27%), ER (14%), men’s medical wards (11%), female surgical wards (10%), and other units with lower isolation rates.

## 4. Discussion

There have been drastic epidemiological changes in *S. aureus* lineage evolutionary patterns, resulting in new strains with enhanced virulence in health care systems globally as well as in this country. New distinct lineages constantly emerge and transmit between both human and animals, crossing all host-species barriers. In the Kingdom of Saudi Arabia, MRSA has been reported as one of the common superbugs associated with severe public health infections [55,75,76,77]. We attempted to address the enhanced virulence and epidemicity in the context of host-selection, resistance, and transmission dynamics. However, there is a severe paucity of high-quality data about the profiles of circulating lineages and demographic patterns. Our data depicted a change in diversity and frequencies of HA-MRSA and MRSA (CA-MRSA) in the northern-central regions of Saudi Arabia. There are several other studies in the region and neighboring countries reporting on the sporadic evolution of community lineages with no clear epidemic profiles [77,78,79,80,81,82]. This potentially relates to the socio-economic migration across Saudi Arabia as a major global economic hub and as well as a holy shrine for pilgrimage. Thus, it has become imperative to understand the reasons behind enhanced virulence associated with specific age groups and the frequency of MRSA profiles across different age- and gender-specific groups in this study to substantiate selective susceptibility among the human host population structure.

In the current study, we show the replacement of HA-MRSA by the CA-MRSA lineages particularly in younger age groups in the Ha’il region of Saudi Arabia in comparison to regional variations. The MRSA isolated from KKH showed an extreme resistance against beta lactams, including amoxicillin–clavulanate and oxacillin at a rate of 83% and nearly absolute resistance against cefoxitin (100%), penicillin (98.6%), and ampicillin (98.6%). More important is that a significantly higher number of isolates developed high resistance (82%) to the commonly used imipenem antibiotic. At the same time, there was a high susceptibility to non-beta lactams, often reaching full susceptibility, such as linezolid (99.3%), teicoplanin (98.6%), rifampicin (98.6%), daptomycin (97.9%), vancomycin (97.3%), nitrofuran (96.6%), and mupirocin (96.6%). These findings indicate the dominance of CA-MRSA lineages in the hospitals consistent with our isolate characterizations. The antibiotic susceptibility profile observed in this study is in line with other studies [82,83]. All CA-MRSA isolates reported in this study were highly susceptible to linezolid, teicoplanin, and vancomycin, which is considered a last resort in MRSA treatment; this stands in agreement with other studies. Hence, this drug should not be used as empirical therapy in the treatment of Gram-positive bacterial infections except in extreme cases. Fortunately, in this study high susceptibility was reported towards TGC, TEI, and SXT. In addition, while there is a prevalence of beta-lactam-resistant MRSA, there is a higher frequency of non-beta lactam susceptibility to gentamicin, erythromycin, and fusidic acid; 33%, 34%, and 38%, respectively, was found. These findings implied that the overwhelming majority (80%; *n* = 119/148) of MRSA identified were CA-MRSA, while only 20% were HA-MRSA. Nevertheless, this contrasted with the scenario in the western regions a decade ago where CA-MRSA infections represented 31.5% of community *S. aureus* infections, while HA-MRSA accounted for 52.6% of hospital *S. aureus,* which was mainly associated with seniors (*p* = 0.0029) [83]. However, an eleven year (2009–2019) surveillance reported an increasing HA-MRSA trend (5% to 14%) among 1338 isolates in the eastern regions, consisting mainly of skin and soft tissue, with a significant reduction in susceptibility to clindamycin (*p* = 0.003), trimethoprim-sulfamethoxazole (*p* = 0.001), and rifampin (*p* < 0.0001) [84]. Unfortunately, data on age and gender specificity of CA-MRSA lineages in different regions for comparison are limited.

An important finding in this study was the age-specific gradual evolutionary replacement of HA-MRSA by the CA-MRSA lineages irrespective of gender differences. This is supported by several findings; for instance, in Table 1, while at the young age group 0 to 20 both adults and children had similar low MRSA counts (MCH *n* = 23; KKH *n* = 19), a significant shift in the MRSA increasing load showed at the age group 21 to 50, with (*n* = 41) from KKH and only seven from MCH. This increasing shift continued, reaching its maximum at the age group >50 with 49%, which is the sum of isolates in the two previous young groups. Intriguingly, despite the significant difference between male (59.2%; *n* = 87) and female (41%; *n* = 60) counts in KKH, as well as the overall 10% difference between genders (male 53%, female 43%), the highest HA-MRSA acquisition point remained age-specific around 58–60 years irrespective of gender differences, as shown in Figure 3a. On the other hand, CA-MRSA initiates early in life and continues to replace the former lineage. These findings have established a solid foundation in support of the established understanding that only a handful of subtle evolutionary mechanisms are involved in the emergence of CA-MRSA epidemics at a young age and HA-MRSA later, apart from resistance. These include host microenvironmental changes at different age-groups that differentiate and select emergence of best fit lineages. While acquired resistance increases epidemicity, it does not significantly alter the inherent adaptive evolution of virulence per se. We and others have established previously that host-specificity, despite genome clonality, is the key in the acquisition of *S. aureus* lineages [8,85]. This strongly supports the notion that fitness in a specific host is usually reached through processes of cellular differentiation and/or the acquisition of lineage-specific trait(s) [10,86]. Genome sequencing revealed variations in a single cohesive population, showing changes in only 30 SNP and four indels accumulating at a steady rate over a 13-mo period, except for a specific cluster of eight mutations preceding the transition to disease that made the new bloodstream variant [87]. Although host-specificity related to age is evident in this study, it is still imperative to understand the original sources of these isolates since *S. aureus* from animals and humans has similar population genetic structures, permitting cross-host transmissions.

The nature of isolates, patient demographics, infection types and sources, and antibiograms all imply novel evolutionary mechanisms in the emergence of CA-MRSA lineages, consistent with previous research. Unfortunately, the bias towards sequencing human-MRSA has created several gaps that hinder understanding of human- and livestock-associated lineages. The CA-MRSA lineage virulence and transmission dynamics is a multifaced mechanism involving the increased use of beta lactams and other antimicrobials in hospitals, agriculture, livestock, and domestic animals. This allows for zoonotic and anthroponoses transmission of animal lineages that are by nature methicillin-sensitive [4,8,9] and has led to the evolution of community-associated *S. aureus* (CA-MRSA) lineages with enhanced virulence in young and otherwise healthy individuals. As a result, waves of global pandemics with significant economic and public health losses have occurred [30,31,32,33,34,35,36,37,38,39]. Many of the reasons for the emergence of virulence were due to the low-level usage of antimicrobial substances [43,44]. For instance, the increased past usage of beta-lactam antibiotics is potentially behind the enhanced virulence which has recently been found to be controlled by the *sarA* gene family [30]. We have conducted a genome-wide expression of dairy and human isolates (unpublished data) under mammary-mimicked hypoxic conditions and revealed specific host factors such as nutritional and glucose impulse, consistent with others [88], and host-tissue specific adherence factors [89,90] that precisely selected bovine-mammary lineages. These factors were controlled by *agr* induction through the environment-sensor, the DNA binding protein of the SarA family, consistent with earlier descriptions [91]. This shows that the intracellular microenvironment of the host cell conditions the emergence of toxigenic strains while repressing protein A (*spa*) [89], a property that distinguishes human and animal lineages, making it imperative to test these isolates in the future for zoonotic transmission potentials.

## 5. Conclusions

We reported on the frequency of CA-MRSA and HA-MRSA infection rates in hospitals with respect to disease patterns, patient demographics, transmission dynamics, and antibiogram patterns. We further identified a potentially host-specific selective replacement of HA-MRSA lineages by the CA-MRSA. Furthermore, irrespective of gender, the acquisition of HA-MRSA infection is largely age-dependent, occurring predominantly around 58–60 years, while CA-MRSA starts early in life and continues to dominate patients, with the most striking disease profiles of this lineage in most patients being the enteritis and necrotizing pneumonia profiles. The prevalence and mechanisms of the emergences of CA-MRSA and MRSA lineages entail a highly complex, orchestrated evolutionary convergence of enhanced virulence, high-resistance, and low-susceptibility to non-beta lactams, in agreement with others [41,42,43]. In contrast, high levels of multi-drug resistance in HA-MRSA lineages confined to hospitals have been associated with mortalities due to hospital-acquired bacteremia, in agreement with others [44]. At present, the exchange of these lineages between the community, hospitals, and livestock animals [45] has made it difficult for planning and clinical management strategies. The most striking difference in these lineages is their patterns in response to treatment options and their acquisition of human-specific markers such as spa, *clfB,* and *clfA*—a property that accurately distinguishes human and animal lineages, making it imperative to test these isolates in the future for zoonotic transmission potentials. This study is limited by the confined area of the two hospitals; the inclusion of other hospitals in the neighboring cities as well as nationwide would have allowed for more insights into *S. aureus* infection patterns to be gained. In addition, a comparative analysis with reference to veterinary strains would have been useful; however, the specific local strain profiling that is necessary is available only in remote regions and therefore it carries the risk of regional differences.

## Figures and Tables

**Table 1:** Patients’ demographics, specimen types, and hospital wards of *Staphylococcus isolates* recovered from Hospitals in Ha’il, Saudi Arabia.

Patient Age	MCH	KKH	Totals	Frequency
0 to 20	23 (70%)	19 (13%)	42	23%
21 to 50	7	41 (28%)	48	27%
>50	2	87 (59%)	89	49%
Not mentioned	1	1	2	
Total	33 (76% CA-MRSA, *n* = 25)	148 (80% CA-MRSA, *n* = 119)	181	80% overall CA-MRSA, *n* = 144
Gender			Total	
Male	15	87	102	70%
Female	18	60	78	53%
Not mentioned	0	1	1	
Total	33	148	181	
Ward KKH (*n* = 148)			Total	
ICU		40	40/148	27%
Men surgical ward		16	16/148	11%
Female surgical ward		15	15/148	10%
Female medical ward		6	6/148	4%
Medical neuropathy		1	1	
Men medical ward		16	16/148	11%
Alkabtonuria		7	7/148	5%
ER		21	21/148	14%
PSW		2	2	
PIW		1	1	
Pediatric specimens	33		33	
Total	33	148	181	

## Data Availability

There is no additional data deposited on any other site other than those in this manuscript.
